# Microlaryngoscopic surgery for pyriform sinus fistulas in children: a report of two cases

**DOI:** 10.1186/s40792-018-0521-5

**Published:** 2018-09-10

**Authors:** Akiyoshi Nomura, Koji Fukumoto, Masaya Yamoto, Toshiaki Takahashi, Kengo Nakaya, Akinori Sekioka, Yutaka Yamada, Naoto Urushihara

**Affiliations:** Department of Pediatric Surgery, Shizuoka Children’s Hospital, 860 Urushiyama, Aoi-ku, Shizuoka City, Shizuoka Prefecture 420-8660 Japan

**Keywords:** Pyriform sinus fistula, Microlaryngoscopy, Children

## Abstract

**Background:**

A pyriform sinus fistula (PSF) is a rare branchial anomaly that causes recurrent cervical infections. Open neck surgery has widely been accepted as a definitive treatment for PSFs, and endoscopic surgery has been reported in recent years. However, both approaches are not satisfactory because of high recurrence rates and postoperative complications. Microlaryngoscopic surgery (MLS) is a transoral surgical technique involving the use of an operating microscope. In this report, we present a new procedure involving MLS for resection and closure of a PSF without a skin incision.

**Case presentation:**

Technique: MLS was performed under general anesthesia with endotracheal intubation. The patient was placed in the supine position, and a direct laryngoscope was inserted to expose the pyriform sinus, which was then magnified using an operating microscope. The mucosal layer was carefully resected using scissors and cupped forceps with sharp edges. The fistula was securely sutured using absorbable suture material. Case 1: A 9-year-old boy with recurrent neck abscesses since 8 years of age presented to our hospital after receiving antibiotics and undergoing drainage in other hospitals. After admission to our hospital, barium esophagography and oral contrast coronal computed tomography showed a PSF on the left side, and open neck surgery was performed to resect the fistula. He was eventually discharged from the hospital without any problem. However, a PSF recurred 2 weeks later. As reoperation with the cervical approach was considered difficult owing to severe adhesions, we adopted MLS as a definitive operation. The postoperative course was uneventful. No recurrence was observed during an 18-month follow-up. Case 2: A 10-year-old girl presented to our hospital with recurrent left-sided neck swelling since 6 years of age. After inflammation control, a PSF was identified on the left side on barium esophagography and computed tomography. MLS was performed safely, and the postoperative course was uneventful. No recurrence was observed during a 10-month follow-up.

**Conclusions:**

MLS allows excellent visualization and effective closure for PSFs, and this approach is suitable for recurrence after open neck surgery. Therefore, MLS might become a first-line treatment for PSFs in children.

## Background

A pyriform sinus fistula (PSF) is a rare congenital anomaly caused by incomplete obliteration of the third or fourth pharyngeal bursa during the seventh week of gestation [1, 2]. A recent theory about the origin of this malformation has suggested that it is derived from the thymopharyngeal duct [2–4]. The chief clinical presentations include recurrent cervical abscesses and suppurative thyroiditis, which are predominantly left sided [5, 6]. The diagnosis of a PSF is difficult because most patients have a clinical history of recurrent cervical abscesses and have already undergone some treatment for these abscesses [7].

The primary treatment for a PSF accompanied by acute cervical infection involves antibiotic administration and surgical abscess drainage. After inflammation control, a definitive treatment is planned. Although open neck surgery for fistula resection has widely been accepted as a definitive treatment, it is sometimes difficult to identify the fistula owing to severe adhesions associated with recurrent infection or repetitive drainage treatments, and postoperative complications subsequently occur.

Several endoscopic techniques have recently been developed to obliterate a PSF, and these have advantages with regard to the identification of the PSF orifice and reduction of postoperative complications [8]. Most reports have mentioned chemical or electrical cauterization for the sinus tract; however, these approaches sometimes result in complications and recurrence. There are few reports on endoscopic resection and closure of a fistula in children. Here, we present a new procedure involving microlaryngoscopic surgery (MLS) with an operating microscope for resection and closure of a PSF without a skin incision.

## Case presentation

### MLS technique

**Fig. 1:**
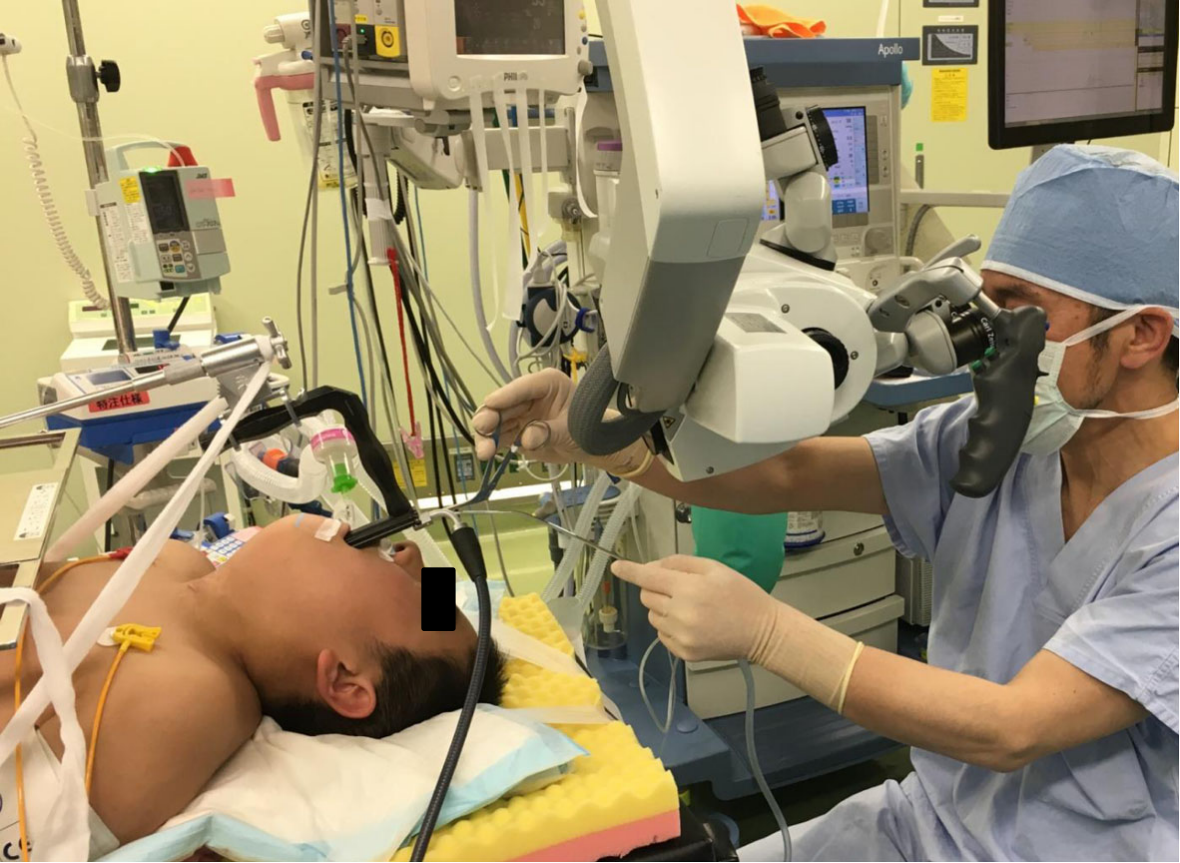
Set-up for microlaryngoscopic surgery

MLS is a common laryngeal operation involving the use of an operating microscope. Surgery is performed under general anesthesia with endotracheal intubation. First, the patient is placed in the supine position, and a direct laryngoscope is inserted to expose the pyriform sinus, which is magnified using an operating microscope (Fig. 1). The fistula orifice is clearly visible in the binocular view. Second, a circumferential incision is made around the internal orifice using scissors and cupped forceps with sharp edges, and the fistula wall is dissected away from the submucosal tissue. Finally, the internal orifice is securely sutured with a single layer of 4–0 Vicryl® using a 13 mm needle and a knot pusher.

### Case 1

**Fig. 2:**
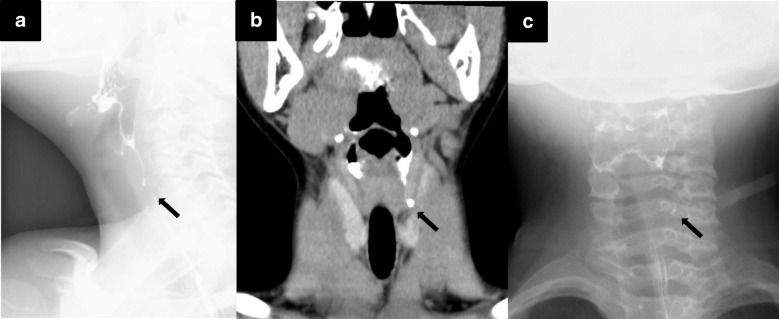
**a** Barium esophagography shows a pyriform sinus fistula (arrow) on the left side. **b** Oral contrast coronal computed tomography shows a fistula (arrow). **c** Barium esophagography after open neck surgery shows recurrence of the pyriform sinus fistula (arrow)

**Fig. 3:**
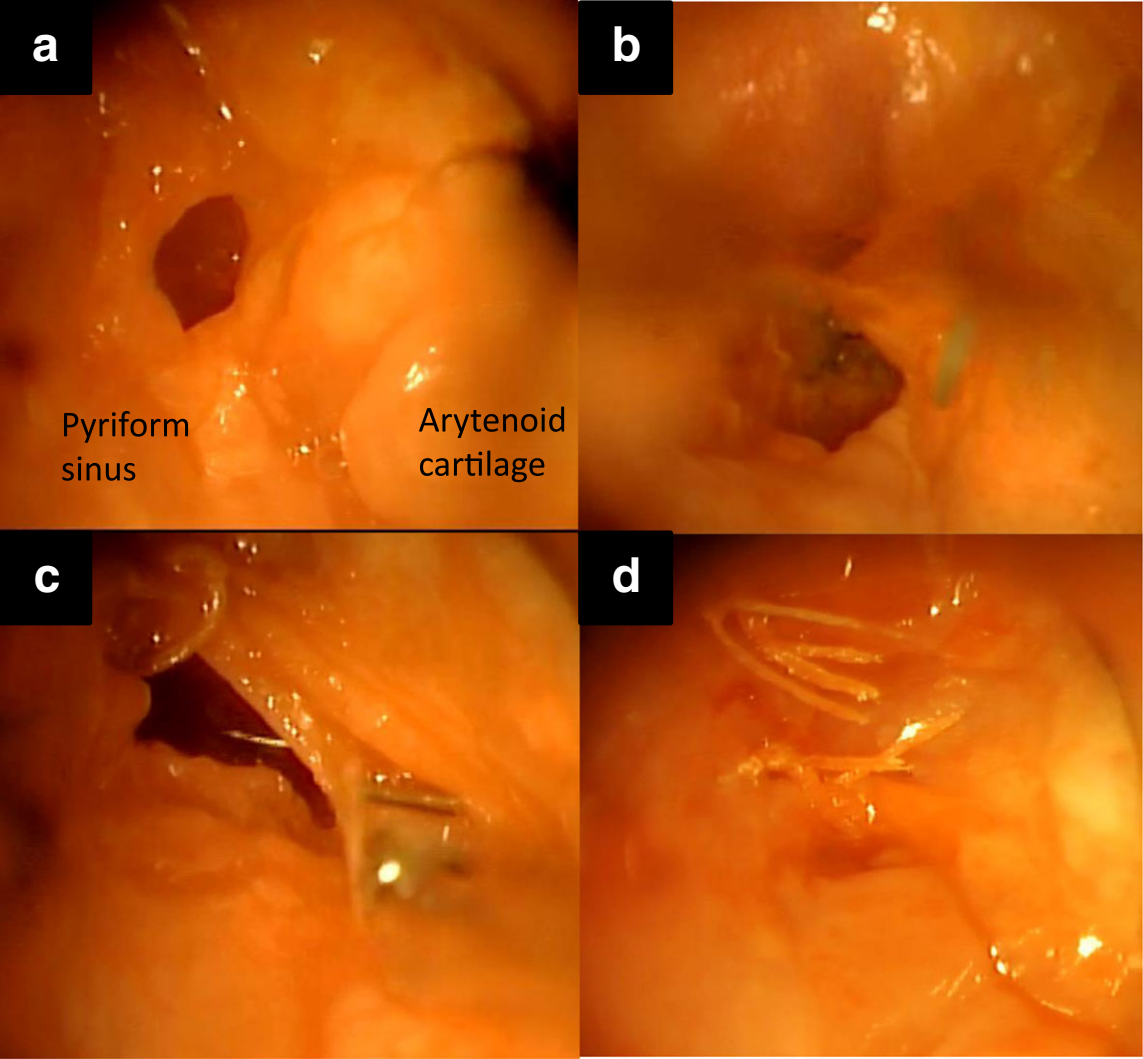
**a** Direct laryngoscopy shows the internal orifice at the left pyriform sinus. **b** The internal orifice is fragile owing to inflammation. A circumferential incision is made around the internal orifice using scissors and cupped forceps. **c** The mucosal incision is sutured using 4–0 Vicryl®. **d** The pyriform sinus after suturing

A 9-year-old boy presented to our hospital with a history of recurrent neck abscesses since 8 years of age. He had received antibiotics and had undergone drainage of the abscesses in other hospitals. He was admitted to our hospital after control of inflammation. Barium esophagography showed a PSF on the left side (Fig. 2a). Oral contrast coronal computed tomography (CT) showed an air- and barium-containing fistula (Fig. 2b). He underwent open neck surgery for definitive treatment of the PSF. He quickly recovered, and he was discharged from the hospital 7 days after the surgery. However, 2 weeks later, he visited our hospital again because of a neck abscess. He received antibiotics and underwent drainage. Barium esophagography revealed fistula recurrence at the same location (Fig. 2c). As reoperation with the cervical approach was expected to be difficult owing to possible severe adhesions, MLS was planned. Although the internal orifice was detected easily (Fig. 3a), the fistula was found to be wider and deeper than expected after resection of the fragile layer associated with inflammation (Fig. 3b). The fistula was resected piecemeal because it could not be easily inverted and peeled off. The entire mucosal remnant was macroscopically removed. Although suturing was difficult because of the wide internal orifice, the procedure was completed uneventfully (Fig. 3c, d). Barium esophagography was performed on the seventh postoperative day, and no issues were noted. He had an uneventful recovery, and he was discharged 10 days after the surgery. No recurrence was observed during an 18-month follow-up.

### Case 2

**Fig. 4:**
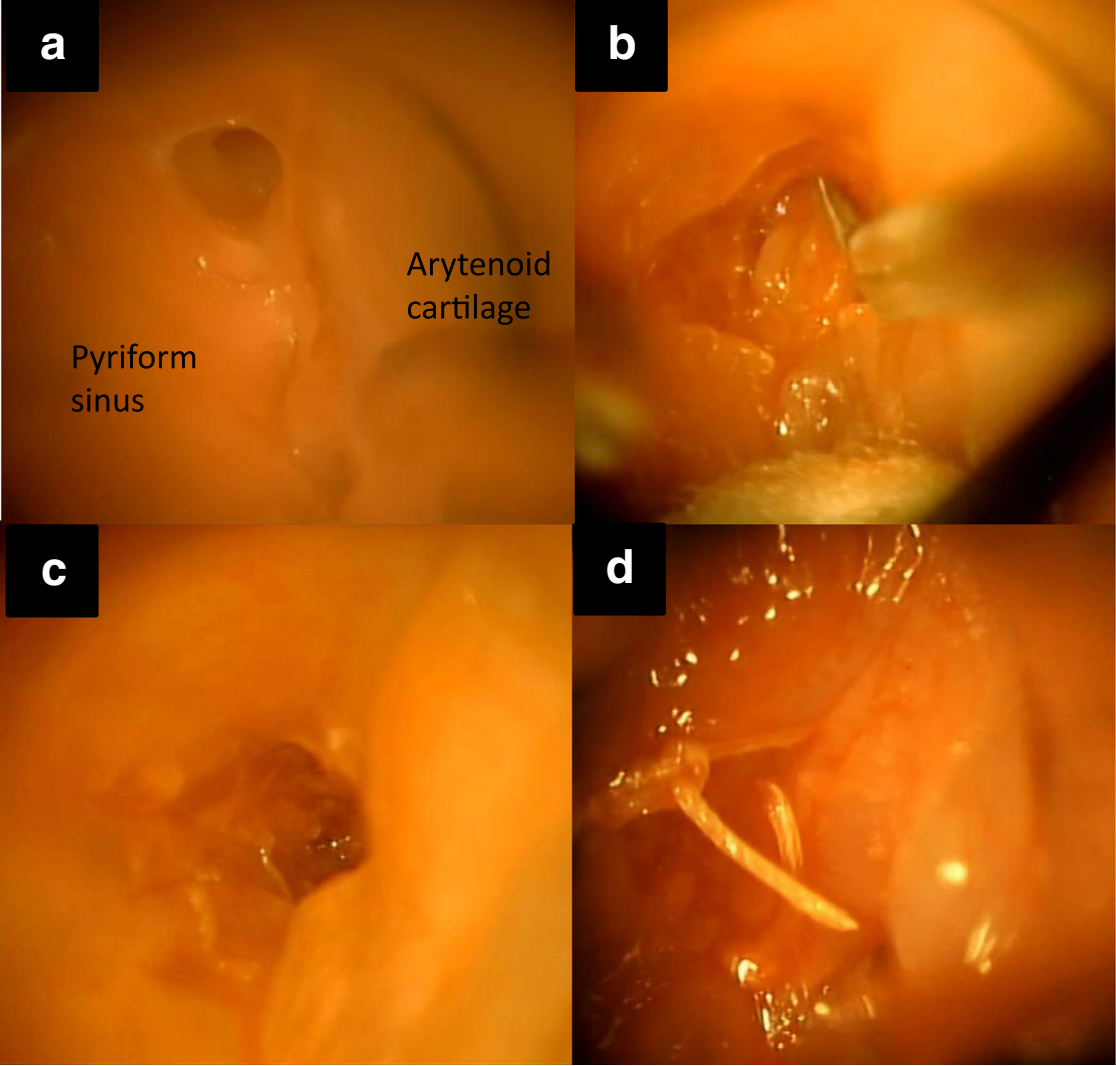
**a** The internal orifice of the fistula is clearly visible using forceps. It is narrow and durable. **b** After making an incision around the orifice, the mucosal layer is dissected using scissors. **c** The fistula after complete mucosal resection. **d** The pyriform sinus after suturing

A 10-year-old girl presented to our hospital with a history of recurrent left-sided neck swelling since 6 years of age. After control of inflammation, barium esophagography was performed, and it showed a PSF on the left side. CT showed an air- and barium-containing fistula. MLS was performed as first-line treatment. The fistula was narrow, and its tissue was not fragile (Fig. 4a). Therefore, we made the incision as small as possible (Fig. 4b, c), and the operation was completed uneventfully (Fig. 4d). Barium esophagography was performed on the fifth postoperative day, and no leakage was noted. Free oral intake was started on the same day. She had an uneventful recovery, and she was discharged 7 days after the surgery. No recurrence was observed during a 10-month follow-up.

### Discussion

A PSF consists of a thin, fragile membranous tract that is closely associated with important structures, such as the recurrent laryngeal nerve, and with acute inflammation, and surrounding scar tissue is always an issue. The definitive treatment for a PSF involves complete excision of the fistula and closure of the internal orifice. Open neck surgery is the most reliable and commonly used procedure. However, the open neck surgical procedure is sometimes difficult because of the anatomical and histological characteristics of the fistula. In addition, adhesions associated with recurrent infection and repetitive drainage could make this procedure difficult [9] and the procedure has some risks such as vocal cord paralysis. Moreover, infection often recurs when the fistula is not completely excised [10]. In a review of 177 published reports on PSFs, among 377 patients who underwent open neck surgery, the recurrence rate was 15% after the initial procedure. Furthermore, the complication rate was 10% in patients who were aged 8 years or younger [7]. Vocal cord paralysis was the most common complication. Higher rates of complications in young children might be associated with anatomical factors and technical difficulties in open surgical excision.

Endoscopic techniques have been developed as alternatives to open neck surgery for PSFs to avoid surgical complications [2, 3, 5, 11]. The most widely used procedure is endoscopic electrocauterization of the fistula tract, which was first described in 1998 [12]. Laser cauterization and chemical cauterization using trichloroacetic acid or silver nitrate and fibrin have also recently been reported [13–15]. Endoscopic approaches have been reported to be less invasive and have better cosmetic outcomes when compared with open neck surgery. However, endoscopic surgery is not satisfactory for PSFs. Approaches involving chemicals might cause tissue inflammation and edema of the nerve, leading to temporary paresis. Vocal fold immobility after endoscopic chemocauterization has been reported in two cases [16]. In addition, chemocauterization has a risk of chemical leakage into the esophagus, which can result in strictures [17]. Moreover, electrocauterization has a risk of pyriform sinus mucosa penetration, which can lead to further abscess formation [18]. Although laser cauterization has the advantage of a thermal effect with less penetration [19], there is no evidence for the superiority of electrocauterization, laser cauterization, or chemocauterization [13]. It has been speculated that endoscopic procedures might retain epithelial remnants of a PSF after cauterization of the tract [3], which can cause recurrence.

Recently, Kamide et al. [20] reported that transoral videolaryngoscopic surgery was effective for adult PSFs. Their procedure avoided recurrence as it involved resection without remnant mucosa and closure of the internal orifice. MLS with an operating microscope is a common transoral approach in laryngeal surgery. MLS can magnify the working space sufficiently in the binocular view, which can ensure mucosal resection and internal orifice closure through direct manipulation in children. Therefore, we believe that MLS for PSFs can help avoid severe complications and recurrence. However, MLS has some limitations. First, en bloc resection of the fistula is often technically challenging because of the limited space. Moreover, the infectious fistula may not be easily inverted and peeled off, and there is a possibility of en bloc resection not being successful. Second, when the PSF is very large, all fistula tracts may not be visible. Because PSFs in children are sometimes large, resection should be carefully performed. Therefore, complete resection is important so that the entire fistula can be sufficiently confirmed via microlaryngoscopy. Although en bloc resection is ideal for fistula, it is challenging because of such limitations. Conversion to piecemeal resection is also useful in difficult cases. Complete resection could be performed via piecemeal resection, and our patient was successfully treated using MLS. Conversely, in case of very large cyst that cannot be completely removed even via piecemeal resection or when a fistula tract cannot be grasped properly, MLS is not considered as first-line treatment for PSF. We previously reported that MLS can be applied in infants aged less than 1 year [21], and we believe that a similar procedure can be performed for PSFs in young children.

Although there are many MLS cases, the number of PSF cases is still small. More PSF cases should be collected, and further assessments of MLS for PSFs should be performed.

## Conclusions

MLS allows excellent visualization and effective closure of PSFs. Moreover, the procedure is suitable for recurrence after open neck surgery. Therefore, MLS can be considered as a safe and minimally invasive procedure, and it might become a first-line treatment for PSFs in children.

## Abbreviations

CT: Oral contrast coronal computed tomography
MLS: Microlaryngoscopic surgery
PSF: Pyriform sinus fistula
